# Electroencephalography-Based Emotion Recognition Using Auditory Stimulation for Affective Brain–Computer Interfaces

**DOI:** 10.3390/s26154971

**Published:** 2026-08-05

**Authors:** Charoenporn Bouyam, Nannaphat Siribunyaphat, Si Thu Aung, Yunyong Punsawad

**Affiliations:** 1 School of Informatics, Walailak University, Nakhon Si Thammarat 80160, Thailand; charoenporn.bo@wu.ac.th (C.B.); nannaphat.sir@mail.wu.ac.th (N.S.); 2 Informatics Innovative Center of Excellence, Walailak University, Nakhon Si Thammarat 80160, Thailand; 3 Division of Mathematics and Science, Parami University, Washington, DC 20036, USA; sithuaung1@parami.edu.mm

**Keywords:** brain–computer interface, electroencephalography, emotion recognition, auditory stimulation, discrete wavelet transform, few-shot adaptation

## Abstract

Although electroencephalography (EEG)-based emotion recognition is a promising approach for affective brain–computer interface (BCI) applications, substantial inter-subject variability continues to limit its generalizability. This study proposed an EEG-based framework to recognize emotions within a valence–arousal model using auditory stimulation. Predefined emotional states were established using validated affective video clips and subsequently evaluated through EEG responses elicited by instrumental melodies. Three EEG features, which include discrete wavelet transform (DWT), functional connectivity (FC), and effective connectivity (EC), together with their combined feature set, were systematically evaluated using five machine learning classifiers. Performance was assessed under subject-dependent, subject-independent (leave-one-subject-out, LOSO), and few-shot subject-adaptation protocols. The results showed that DWT achieved the highest subject-dependent classification accuracy (0.88), followed by the combined feature set (0.84). In contrast, the subject-independent LOSO evaluation yielded near-chance performance across all feature domains (0.23–0.30), highlighting substantial inter-subject variability. Few-shot subject adaptation using 25–75% subject-specific calibration data substantially improved subject-independent performance, with the highest accuracy of 0.77 achieved by FC at 75% calibration. In conclusion, these findings demonstrate the feasibility of EEG-based emotion recognition using auditory stimulation under predefined affective conditions and provide a foundation for the future development of personalized affective brain–computer interface systems.

## 1. Introduction

Emotion recognition is a critical area of research in healthcare, rehabilitation, and human–computer interaction because emotional states influence communication, behavior, and overall well-being [1,2,3]. Emotions are commonly represented using discrete emotion categories (e.g., happiness, sadness, fear, and anger) or dimensional models. Among these, the valence–arousal model is widely adopted because it characterizes emotional states along two continuous dimensions: emotional valence (pleasant–unpleasant) and arousal (high–low activation). This dimensional framework has been widely used in biosignal-based emotion recognition. Objective assessment of emotional states is particularly valuable in clinical settings where patients are unable to communicate their feelings effectively, such as individuals with communication impairments, those receiving palliative care, and patients with disorders of consciousness or reduced levels of consciousness [4,5,6]. In these populations, conventional emotion assessment based on facial expressions, body movements, or verbal responses may be unreliable or infeasible because behavioral responses are often limited or absent. Consequently, there is a growing need for objective approaches that can infer internal emotional or affective states directly from physiological signals. Patients with disorders of consciousness often undergo repeated behavioral assessments and supportive interventions, including sensory stimulation, to promote awareness and responsiveness [7,8,9,10,11]. However, these approaches provide limited information about patients’ internal affective states. Therefore, a system capable of detecting neural responses associated with emotional processing directly from brain signals, without relying on overt communication or observable behavior, could provide complementary information to support personalized patient monitoring and rehabilitation.

Several neurophysiological techniques, including electroencephalography (EEG), functional magnetic resonance imaging (fMRI), functional near-infrared spectroscopy (fNIRS), and magnetoencephalography (MEG), have been used to investigate the neural mechanisms underlying emotional processing. Among these, EEG is a noninvasive, relatively low-cost technique with high temporal resolution for recording brain electrical activity, making it particularly suitable for monitoring the rapid neural dynamics associated with emotion processing [12,13]. Compared with behavioral or peripheral signals such as facial expressions, speech, heart rate, or skin conductance, EEG can directly reflect central nervous system activity and is difficult for participants to manipulate or feign [14,15,16]. Consequently, EEG-based emotion recognition has gained significant attention in affective BCI research, where it provides a foundation for developing systems capable of recognizing users’ emotional states and adapting system behavior accordingly [17,18,19].

Most EEG-based emotion recognition studies use external stimuli, such as images, videos, sounds, music videos, or songs, to elicit emotional responses. Each stimulus type has distinct characteristics, and videos are widely used because they integrate visual, auditory, and contextual information to induce emotional states [20,21,22]. Previous studies have examined a range of EEG feature representations and machine learning methods for emotion recognition. For example, Li et al. combined differential entropy-based EEG topographic maps with facial expression images to classify emotions and reported changes in the prefrontal, temporal, and occipital brain regions during emotional processing [23]. Wang et al. extracted functional connectivity features using phase-locking value (PLV) and persistent homology from the DEAP, SEED, and DREAMER datasets for emotion recognition [24]. Cui et al. constructed PLV-based adjacency matrices and used a convolutional neural network (CNN) to classify valence and arousal, comparing individual and group EEG representations [25]. Akhand et al. combined multiple connectivity measures, including Pearson correlation coefficient (PCC), PLV, mutual information (MI), and transfer entropy (TE), for CNN-based emotion classification [26].

In addition to videos, music has also been widely used as an emotion-elicitation stimulus because it can evoke emotional responses over time. Wei et al. investigated EEG responses to traditional Chinese music in patients with major depressive disorder and healthy controls using directed functional connectivity analysis [27]. Zhou et al. proposed EEG- and music-based models for emotion classification in the context of music therapy [28]. Bo et al. analyzed temporal changes in EEG responses before, during, and after music listening to investigate emotion dynamics [29]. These studies demonstrate that EEG features derived from time–frequency analysis, functional connectivity, and effective connectivity have been investigated for emotion recognition under different experimental paradigms [30,31,32].

A detailed comparison of representative EEG-based emotion recognition studies, including their experimental paradigms, feature representations, classification methods, validation protocols, and reported performance, is presented in Section 4. While previous studies have investigated a variety of emotion-elicitation stimuli, EEG feature representations, and classification methods, the present study examines EEG responses to auditory melody stimulation following video-induced emotional states. Specifically, emotional states are first induced with validated video stimuli and then assessed via EEG responses to auditory melody stimulation. The proposed framework classifies four emotional quadrants—high valence–high arousal (HVHA), high valence–low arousal (HVLA), low valence–high arousal (LVHA), and low valence–low arousal (LVLA)—using multiple EEG representation domains, including time–frequency, functional connectivity, and effective connectivity features. Their performance is systematically evaluated under subject-dependent, subject-independent, and calibration-based subject-adaptation protocols.

The objective of this study is to systematically evaluate different EEG representation domains for auditory emotion recognition and their performance under subject-dependent, subject-independent, and calibration-based subject-adaptation scenarios. As a proof of concept, the framework was evaluated under controlled laboratory conditions with healthy participants to establish feasibility before future validation in clinical populations. The main contributions of this study are summarized as follows:

1. An experimental paradigm that establishes predefined emotional states using validated video clips before auditory stimulation is proposed to investigate EEG-based auditory emotion recognition within the valence–arousal model.
2. Three EEG representation domains—time–frequency, functional connectivity, and effective connectivity—are systematically compared under identical experimental conditions.
3. Subject-dependent, leave-one-subject-out (LOSO), and calibration-based few-shot subject-adaptation protocols are evaluated to investigate subject-specific and cross-subject performance.
4. The effectiveness of different EEG representation domains is evaluated for subject-dependent, subject-independent, and subject-adaptive auditory emotion recognition.

## 2. Materials and Methods

**Figure 1:**
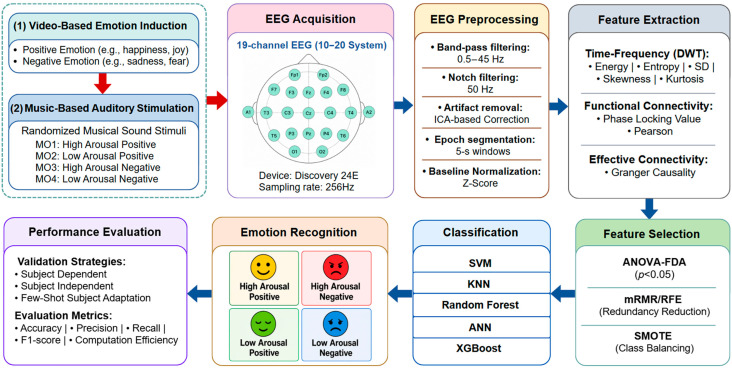
Overall framework of the proposed EEG-based auditory emotion recognition system.

The proposed methodology was developed to investigate EEG responses to auditory stimulation under predefined emotional states within the valence–arousal dimensional model. As illustrated in Figure 1, the framework consists of seven sequential stages: (1) emotion induction using validated affective video clips; (2) auditory stimulation with instrumental melodies; (3) EEG acquisition; (4) EEG preprocessing; (5) multidomain feature extraction; (6) feature selection and machine learning classification; and (7) performance evaluation.

Initially, participants viewed validated affective video clips to induce predefined emotional states corresponding to the four valence–arousal quadrants. This phase established controlled emotional states before auditory stimulation, providing a predefined affective context for investigating whether EEG responses elicited by musical stimuli could discriminate different emotional states. Immediately afterward, instrumental melodies representing each emotional quadrant were presented while EEG signals were recorded. The auditory stimuli included both emotion-congruent and emotion-incongruent conditions relative to the induced emotional state, enabling the investigation of EEG responses under different affective contexts. EEG signals were recorded during both phases; however, the primary objective of the proposed framework was to determine whether EEG responses elicited during auditory stimulation provide more discriminative information for emotion recognition than EEG recorded during emotion induction.

The proposed framework represents an experimental paradigm for investigating EEG-based auditory emotion recognition under controlled affective conditions. By establishing predefined emotional states before auditory stimulation, the framework enables a systematic evaluation of whether auditory-evoked EEG responses can discriminate previously established emotional states, thereby supporting the development of future real-time auditory stimulus-based emotion recognition systems. During the preparation of this manuscript, the authors used ChatGPT (GPT-5.6, OpenAI) to assist in preparing and refining the figures. The authors have reviewed and edited the output and take full responsibility for the content of this publication.

### 2.1. EEG Acquisition

A total of 25 healthy volunteers (17 males and 8 females) participated in this study. The participants were aged 18–23 years (mean age = 20.76 ± 1.21 years). None of the patients reported any history of neurological or psychiatric disorders, hearing impairment, or use of medications affecting the central nervous system. Each volunteer received a detailed explanation of the procedure and provided written informed consent before participation. The study protocol complied with the Declaration of Helsinki and was approved by the Human Research Ethics Committee of Walailak University (project no. WU-EC-IN-2-018-68, approval no. WUEC-25-085-01; approved on 13 March 2025).

A BrainMaster Discovery-24 amplifier (BrainMaster Technologies, Inc., Bedford, OH, USA) was used to record EEG signals, with 19 electrodes placed according to the international 10–20 system. The recording channels included Fp1, Fp2, F7, F3, Fz, F4, F8, T3, C3, Cz, C4, T4, T5, P3, Pz, P4, T6, O1, and O2, with A1 and A2 as reference electrodes. Electrode impedance was kept below 10 kΩ, and the sampling rate was set to 256 Hz. Initial preprocessing was conducted using the Discovery software (version 1.6.0) to minimize physiological artifacts and environmental noise. A 0.5–40 Hz bandpass filter preserved the relevant EEG frequency bands, including theta, alpha, beta, and low-gamma rhythms, while removing baseline drift and high-frequency noise. A 50 Hz notch filter was applied to suppress power-line interference.

### 2.2. Experimental Stimuli and Paradigm

An experimental paradigm comprising video-based emotion induction followed by auditory stimulation was employed to investigate whether EEG responses elicited by musical stimuli could discriminate predefined emotional states. The protocol was designed according to Russell’s circumplex model of emotion, which characterizes affective states along two orthogonal dimensions: valence (pleasant–unpleasant) and arousal (high–low). Initially, validated video clips were used to induce target emotional states. This emotion induction procedure established a controlled affective context for the subsequent auditory EEG evaluation. Immediately afterward, participants listened to instrumental melodies while EEG signals were recorded to examine brain responses to auditory stimuli under the predefined emotional conditions. EEG signals were recorded during both the emotion induction and auditory stimulation phases to enable comparison between video-evoked and auditory-evoked EEG responses.

#### 2.2.1. Video-Based Emotion Induction and Stimulus Validation

The video clips were used exclusively to induce predefined emotional states according to Russell’s circumplex model of emotion. The emotion induction procedure was designed to establish a controlled affective context before auditory stimulation, enabling the investigation of whether EEG responses elicited by musical stimuli could discriminate different emotional states. Consequently, the video clips were not used as part of the emotion recognition algorithm but solely to establish predefined emotional states for subsequent auditory EEG evaluation. An initial set of 20 candidate videos was collected from publicly available multimedia sources and selected to represent the four affective quadrants: high valence–high arousal (HVHA), high valence–low arousal (HVLA), low valence–high arousal (LVHA), and low valence–low arousal (LVLA), corresponding to happiness, calmness, fear, and sadness, respectively.

A preliminary assessment was conducted with healthy volunteers who did not participate in the EEG experiment to validate the emotional content of the videos. Under controlled laboratory conditions, participants viewed all candidate video clips. They rated their emotional responses using the self-assessment manikin, a nonverbal affective assessment tool that measures perceived valence and arousal on a nine-point scale. The means and standard deviations of valence and arousal ratings were calculated for each video clip. Video clips with high emotional consistency (low standard deviation) and clear separation within the valence–arousal space were selected.

**Figure 2:**
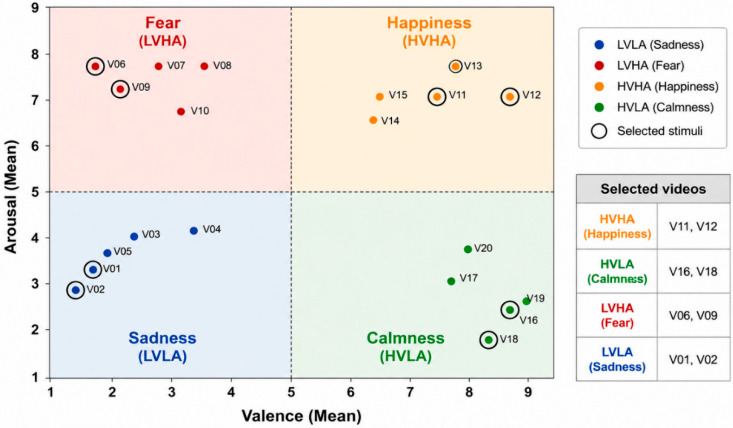
Distribution of the candidate video stimuli in the valence–arousal space based on mean self-assessment manikin (SAM) ratings. Circled markers indicate the video clips selected for the EEG experiment.

Based on the validation results, eight video clips were selected, with two clips representing each affective quadrant. The selected clips provided the predefined emotional contexts for the subsequent auditory stimulation experiment and are summarized in Table 1. The distribution of the validated stimuli within the valence–arousal space is illustrated in Figure 2, where selected clips form four distinct clusters corresponding to the target emotional categories.

#### 2.2.2. Auditory Stimulation

Following video-based emotion induction, auditory stimulation was delivered using instrumental melodies designed according to the same valence–arousal framework. Four melody categories were employed, representing the four affective quadrants of Russell’s circumplex model of emotion: high valence–high arousal (HVHA), high valence–low arousal (HVLA), low valence–high arousal (LVHA), and low valence–low arousal (LVLA). Instrumental melodies without lyrics were selected to minimize semantic processing and ensure that emotional responses were primarily elicited by their acoustic characteristics.

The selected melodies differed in perceptual acoustic attributes, including pitch, loudness, tempo, and timbre, to evoke distinct emotional states. These attributes are commonly characterized using psychoacoustic measures, such as the Mel scale for perceived pitch and the Sone scale for perceived loudness.

The auditory stimuli were presented binaurally through a Sony Soundbar HT-S100F (Sony Corporation, Tokyo, Japan) at an approximately 70 dB sound pressure level (SPL). The playback level was measured using a digital sound level meter (TETSL01) and maintained consistently for all participants throughout the experiment. Participants self-reported normal hearing and no history of hearing impairment or neurological disorders; however, formal audiometric evaluation was not performed.

Each melody was presented for 10 s and repeated three times in a randomized order, resulting in a total stimulation duration of 120 s. Randomization was applied to minimize sequence effects and habituation across trials. The auditory stimulus categories are summarized in Table 2. During auditory stimulation, participants were instructed to remain relaxed, minimize body movements, and attend to the presented melodies while EEG signals were continuously recorded. The combination of validated video-based emotion induction and controlled auditory stimulation was designed to establish stable affective states and facilitate the investigation of EEG responses associated with auditory emotion processing.

### 2.3. Experimental Procedure

The experiment was conducted in two separate sessions on different days to ensure high-quality EEG recordings and reduce participant fatigue. One session was designed to induce positive emotions, whereas the other was designed to induce negative emotions. Each session comprised four trials, which resulted in eight trials per participant. Each session lasted ~60 min.

**Figure 3:**
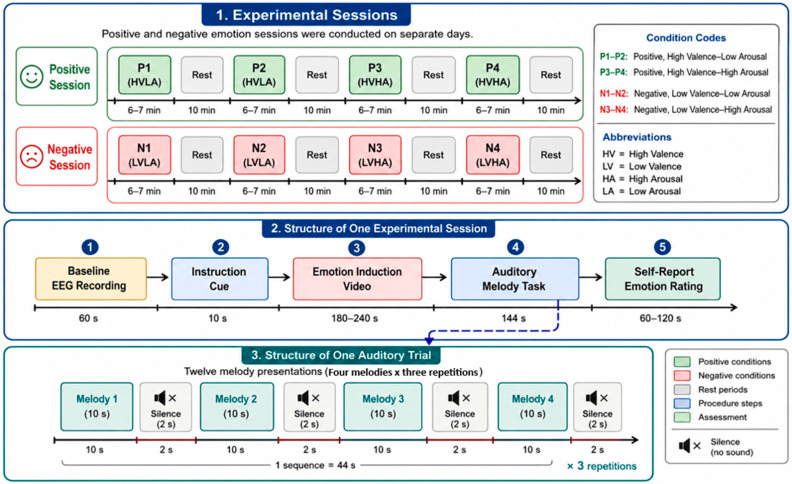
Experimental paradigm used for EEG data acquisition.

As shown in the experimental sessions of Figure 3, the positive-emotion session (P1–P4) consisted of two high valence–low arousal (HVLA) and two high valence–high arousal (HVHA) video trials, whereas the negative-emotion session (N1–N4) consisted of two low valence–low arousal (LVLA) and two low valence–high arousal (LVHA) video trials, as listed in Table 1. A 10 min rest period was provided between consecutive trials to minimize emotional carryover effects and mental fatigue.

The structure of one experimental session section in Figure 3 illustrates the timeline of a single trial. Each trial began with a 60 s baseline EEG recording, during which participants maintained a neutral emotional state while fixating on a cross displayed at the center of the screen. This was followed by a 10 s instruction cue, after which participants viewed an emotion-inducing video clip lasting 180–240 s. Immediately after the video, participants completed an auditory melody task (144 s), followed by a self-report emotion assessment requiring approximately 60–120 s.

Immediately after viewing the video, participants performed the auditory melody task, as illustrated in the structure of one auditory trial section of Figure 3. The task consisted of four categories of instrumental melodies based on Russell’s circumplex model of emotion: HVHA, HVLA, LVHA, and LVLA. Each melody was presented for 10 s, followed by a 2 s silent interval. The four melody categories listed in Table 2 (M01–M04) were repeated three times in a randomized order, resulting in 12 melody presentations and a total auditory stimulation duration of 144 s. Following auditory stimulation, participants completed a self-report emotion assessment by selecting the emotional category (HVHA, HVLA, LVHA, or LVLA) that best matched their current emotional state. The assessment required approximately 60–120 s to complete. Finally, a 10 min rest period was provided before the next trial to minimize emotional carryover effects and mental fatigue.

### 2.4. Auditory EEG Dataset Construction

An auditory EEG dataset was collected from the participants. After each video stimulus, four auditory conditions corresponding to Russell’s circumplex model of emotion (HVHA, HVLA, LVHA, and LVLA) were presented and repeated three times. This protocol produced 96 EEG recordings per participant and 1440 recordings in total. Each auditory stimulation lasted 10 s. Each recording was divided into three overlapping 5 s segments with a 50% overlap (0–5 s, 2.5–7.5 s, and 5–10 s) to create more training samples while maintaining temporal continuity. Consequently, the final dataset comprised 4320 EEG epochs used for feature extraction, feature selection, emotion classification, and few-shot subject adaptation experiments.

Each EEG epoch was assigned emotion and congruency labels to analyze the emotional state and congruency between the auditory stimulus and emotion. The emotion label was assigned according to the verified emotional state established during the preceding video-based emotion induction procedure, and the epoch was classified into one of four emotional groups: HVHA, HVLA, LVHA, or LVLA. Thus, each auditory EEG epoch represented the participant’s EEG response to musical stimulation under a predefined and verified affective context. The second label indicated whether the emotional state of the video matched the auditory stimulus. Auditory stimuli that aligned with the verified emotional state were labeled 1 (matching), whereas mismatched stimuli were labeled 0 (non-matching). For example, an HVHA melody following an HVHA video received 1, whereas an LVLA melody following an HVHA video received 0.

Baseline EEG recordings obtained before emotion induction served as neutral reference data for normalization and spectral analysis; however, these recordings were excluded from the emotion classification dataset, and 4320 EEG epochs were used for model training and testing.

### 2.5. Proposed Algorithms

The EEG signals were preprocessed using band-pass and notch filters, artifact removal, epoch segmentation, and baseline normalization. Features were extracted from three EEG domains: time–frequency, functional connectivity, and effective connectivity. Feature selection used ANOVA-FDA and mRMR/RFE, and SMOTE was used for class balancing. Finally, multiple machine learning algorithms classified the features, and their performance was evaluated using three validation protocols: subject-dependent, leave-one-subject-out (LOSO), and calibration-based few-shot subject adaptation.

#### 2.5.1. Signal Preprocessing

Raw EEG signals were preprocessed using the procedure described in Figure 3. Band-pass filtering (0.5–45 Hz) and notch filtering were applied to remove noise and power line interference. Subsequently, independent component analysis (ICA) was employed to identify and remove physiological artifacts including electrooculogram and electromyogram contamination.

The cleaned EEG signals were segmented into epochs corresponding to the stimulus presentation intervals. For the speech stimuli, segments were extracted based on 0.5 s stimulation windows, whereas 10-s epochs were utilized for the melody stimuli. Baseline correction was applied to each epoch using the pre-stimulus neutral period to ensure that the analyzed neural responses were elicited by auditory stimuli.

#### 2.5.2. EEG Feature Extraction

Three complementary EEG domains were used to extract features characterizing emotional responses to auditory stimulation: time–frequency representation, functional connectivity, and effective connectivity. These domains were selected to capture spectral–temporal changes, inter-regional synchronization, and directed interactions among EEG channels. For the time–frequency domain, the discrete wavelet transform decomposed each EEG epoch into multiple frequency sub-bands while preserving both temporal and spectral information [33,34]. The DWT coefficient at scale j and translation k is defined as

(1) $$W_{x}(j,k)=\sum_{n=0}^{N-1}x\left[n\right]\psi_{j,k}^{*}\left[n\right]$$

where x[n] denotes the EEG signal, N is the number of samples, $\psi_{j,k}\left[n\right]$ is the wavelet basis function at scale j and translation k, and ${\left(\cdot \right)}^{*}$ denotes the complex conjugate. From the wavelet coefficients of each frequency band, statistical features were computed, including signal energy, Shannon entropy, mean, standard deviation, skewness, and kurtosis. Functional connectivity features were extracted using the phase-locking value and Pearson correlation coefficient to quantify phase synchronization and linear association between pairs of EEG channels [24,25]. The PLV between two EEG channels was calculated as

(2) $$PLV=\left|\frac{1}{N}\sum_{n=1}^{N}e^{i\left[\phi_{1}(n)-\phi_{2}(n)\right]}\right|$$

where $\phi_{1}(n)$ and $\phi_{2}(n)$ denote the instantaneous phases of the two EEG channels at sample n, N is the number of samples, and $i=\sqrt{-1}$. The PCC was computed as

(3) $$r_{XY}=\frac{\sum_{n=1}^{N}\left(X_{n}-\bar{X}\right)\left(Y_{n}-\bar{Y}\right)}{\sqrt{\sum_{n=1}^{N}{\left(X_{n}-\bar{X}\right)}^{2}}\;\sqrt{\sum_{n=1}^{N}{\left(Y_{n}-\bar{Y}\right)}^{2}}}\;$$

where $X_{n}$ and $Y_{n}$ are samples from two EEG channels, and $\bar{X}$ and $\bar{Y}$ are their respective mean values. Effective connectivity was estimated using Granger causality to characterize the directional influence between EEG signals [35,36,37]. To determine whether signal X Granger causes signal Y, a restricted autoregressive model using only the past values of Y is defined as

(4) $$Y_{t}=\sum_{i=1}^{p}a_{i}Y_{t-i}+\varepsilon_{r,t}$$

whereas the unrestricted model incorporates the past values of both signals:

(5) $$Y_{t}\;=\;\sum_{i\;=\;1}^{p}a_{i}Y_{t-i}+\;\sum_{i=1}^{p}b_{i}X_{t-i}+\;\varepsilon_{u,t}$$

where $X_{t}$ and $Y_{t}$ represent two EEG time series, p is the autoregressive model order, and $\varepsilon_{r,t}$ and $\varepsilon_{u,t}$ are the residuals of the restricted and unrestricted models, respectively. The GC strength from X to Y was calculated as

(6) $$F_{X\to Y}=In\left(\frac{Var\left(\varepsilon_{r,t}\right)}{Var\left(\varepsilon_{u,t}\right)}\right)$$

A positive value of $F_{X\to Y}$ indicates that including the past values of X reduces the prediction error of Y, suggesting a directed influence from X to Y. The extracted multidomain features were then subjected to feature selection and classification.

#### 2.5.3. Feature Selection and Dimensionality Reduction

The feature selection pipeline for the auditory stimulation dataset followed a robust statistical framework to ensure methodological consistency and facilitate a direct comparison between the two experimental paradigms. The high-dimensional feature space resulting from multidomain extraction across 19 channels was systematically pruned through the following stages.

1. Statistical filtering via one-way analysis of variance with false discovery rate (ANOVA-FDR) [38,39]: ANOVA was initially conducted to evaluate the discriminative power of each feature across the four emotional classes. FDR control using the Benjamini–Hochberg procedure was implemented to account for the multiple comparison problem inherent in large-scale EEG feature sets. Only features that maintained statistical significance (adjusted *p* < 0.05) after FDR correction were retained for further analysis.
2. Redundancy mitigation: The statistically significant feature subset was refined using the minimum redundancy maximum relevance [40] and recursive feature elimination strategies [41]. This ensured that the final feature pool was not only highly relevant to the emotional labels, but also minimized redundant information, enhancing the generalization capability of machine learning models.
3. Handling class imbalance: The synthetic minority oversampling technique [42] was applied to the training data to prevent classification bias. This balanced the representation of the four affective quadrants (HVHA, HVLA, LVLA, and LVHA) and ensured that the resulting models were equally sensitive to all emotional states during the auditory stimulation trials.

#### 2.5.4. Feature Fusion

The DWT, functional connectivity (FC), and effective connectivity (EC) features characterize complementary aspects of EEG activity, including spectral characteristics, undirected functional interactions, and directed information flow. To exploit the complementary information provided by these heterogeneous feature domains, two feature fusion strategies were investigated: direct concatenation and simple fusion.

In direct concatenation, feature vectors from the three domains were merged into a single feature space before feature selection. The combined ANOVA-FDR and redundancy-reduction procedure described in Section 2.5.3 was then applied to the pooled feature set, allowing features from all domains to compete directly based on their discriminative ability. The resulting combined (DWT + FC + EC) feature subset was subsequently used for the subject-dependent, subject-independent (LOSO), and few-shot subject-adaptation experiments. In simple fusion, feature selection was first performed independently within each feature domain, followed by domain-wise standardization and feature concatenation to construct the final feature representation. Both fusion strategies were evaluated using the same feature selection, classification, and evaluation procedures, thereby isolating the effect of the feature fusion strategy on auditory EEG emotion recognition.

#### 2.5.5. Emotion Classification and Validation

The classification of affective states was conducted using the same suite of machine learning models to ensure that any observed differences in performance were attributable to the characteristics of neural responses rather than model-specific bias. The five selected classification algorithms were a support vector machine (SVM) with a radial basis function (RBF) kernel, k-nearest neighbors (KNN), random forest (RF), artificial neural network (ANN), and extreme gradient boosting (XGBoost).

The classification task followed a four-class affective circumplex framework consisting of HVHA, HVLA, LVLA, and LVHA. A three-tier validation strategy described in Section 3.5 was employed to rigorously evaluate model performance and generalizability: 10-fold stratified cross-validation for subject-dependent assessment, leave-one-subject-out (LOSO) cross-validation for subject-independent evaluation, and few-shot subject adaptation for personalized model recovery [27,38,43,44]. The hyperparameter configurations used for each classifier are listed in Table 3.

To further investigate cross-subject adaptation, two representative domain-adaptation methods, correlation alignment (CORAL) and transfer component analysis (TCA), were evaluated alongside conventional few-shot fine-tuning under the LOSO protocol. CORAL reduces domain discrepancy by aligning the covariance statistics of source and target feature distributions, whereas TCA learns a shared latent feature space that minimizes distribution mismatch between source and target domains. Performance was evaluated using progressively increasing amounts of subject-specific calibration data (25%, 50%, and 75%), enabling a systematic comparison of multidomain EEG representations and adaptation strategies.

## 3. Experimental Results

### 3.1. Observation of EEG Response During Video Induction and Musical Auditory Stimulation

**Figure 4:**
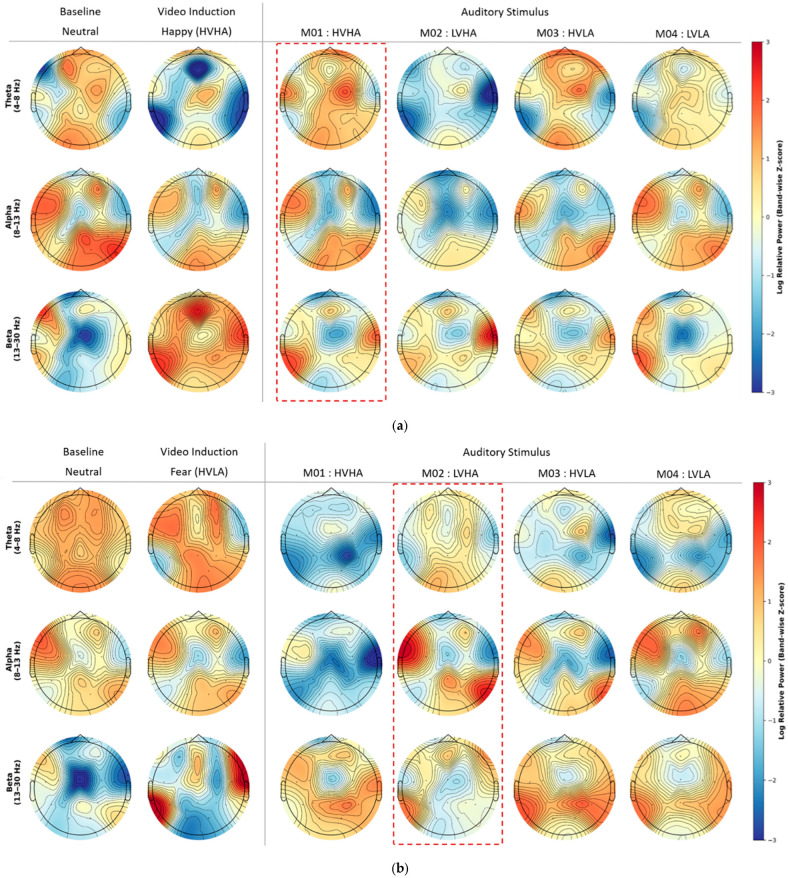
Example of brain topographic maps showing preservation of emotional states during auditory stimulation. (**a**) Positive emotion induction (happiness, HVHA) followed by four auditory conditions. (**b**) Negative emotion induction (fear, LVHA) followed by four auditory conditions. Dashed red boxes mark the auditory condition with the greatest spatial similarity to the corresponding video-induced emotional state across the theta (4–8 Hz), alpha (8–13 Hz), and beta (13–30 Hz) frequency bands.

Figure 4 presents representative EEG topographic maps generated in Python (version 3.13.0) to illustrate the relationship between the video-induced emotional states and subsequent auditory stimulation. The figure focuses on the spatial distribution of EEG activity across frequency bands; therefore, representative EEG waveforms are not included to maintain the clarity of the spatial comparisons. Figure 4a shows a representative positive emotional state (happiness; Subject S001, Video V8), whereas Figure 4b shows a representative negative emotional state (fear; Subject S001, Video V4).

As shown in Figure 4a, the HVHA auditory stimulus (M01) produced topographic patterns that most closely resembled those observed in the happiness-inducing video. Similar activity distributions were observed across the theta, alpha, and beta frequency bands, particularly in the frontal and centroparietal regions [45,46]. This suggests that emotionally congruent auditory stimulation may help maintain the underlying positive affective state. Similarly, as shown in Figure 4b, the LVHA auditory stimulus (M02) most closely matched the EEG patterns observed during the fear induction video. The preservation of the characteristic spatial activity across multiple frequency bands indicates that congruent negative auditory stimulation may reinforce the neural representation of the induced negative emotional state. Observations suggest that auditory stimuli aligned with the emotional state of the preceding video can sustain similar EEG patterns, implying that EEG responses reflect ongoing emotional states during processing.

### 3.2. Feature Dimensionality and Subject-Dependent Performance

The effectiveness of EEG-based emotion recognition depends on feature quality and size. More features can offer richer neural information; however, redundant features increase complexity and reduce generalizability. An optimal feature subset is crucial for accurate emotion classification. This study evaluated multidomain features of time–frequency, functional connectivity, and EC within a subject-dependent framework. Using a ten-fold stratified cross-validation, the classification was evaluated across four feature subsets (top 20, 50, 75, and 100) to determine the optimal dimensionality for auditory emotion decoding.

Table 4 shows classification accuracies across feature subset sizes (top 20, top 50, and top 100) for the DWT, FC, EC, and combined (DWT + FC + EC) domains. Across all domains and classifiers, performance improved consistently with an increasing number of selected features, with the top-100 subset achieving the highest accuracy in every domain. This trend indicates that larger feature subsets retained more discriminative information for emotion recognition, particularly for the DWT and combined feature domains, whereas the FC and EC domains exhibited more gradual improvements. Therefore, we selected the top-100 feature subset for a detailed performance comparison, as shown in Table 5.

Table 5 presents the classification performance of the top 100 features across the four feature domains. In the DWT domain, ANN achieved the highest accuracy of 0.88 ± 0.05, followed by SVM at 0.85 ± 0.05, highlighting the effectiveness of time–frequency features for auditory emotion recognition. Similar precision, recall, and F1-score values indicate consistent and reliable classification across all classifiers. In the FC domain, SVM achieved the highest accuracy of 0.78 ± 0.03, followed by ANN at 0.76 ± 0.03, reflecting a meaningful but comparatively smaller contribution from inter-regional phase synchronization than from time–frequency dynamics. In the EC domain, ANN and SVM achieved the highest accuracy of 0.70 ± 0.03, the lowest among the three single-domain feature sets, suggesting that directional neural flow alone is less informative for distinguishing affective states under auditory stimulation.

The combined (DWT + FC + EC) domain, which fuses all three feature sets, achieved its best performance with SVM at 0.84 ± 0.04 and with ANN at 0.81 ± 0.04, which was competitive with but did not exceed the best single-domain result (DWT). This finding suggests that integrating heterogeneous EEG representations does not necessarily improve classification performance when the additional features provide redundant or less discriminative information. Robustness across all feature domains indicates that both spectral attributes and interregional neural interactions provide meaningful representations for auditory emotion recognition. Furthermore, the close agreement among accuracy, precision, recall, and F1-score across all feature domains indicates balanced classifier performance without substantial bias toward any emotion class.

### 3.3. Subject-Independent Emotion Decoding

Table 6 shows the distribution of LOSO classification accuracies across the DWT, FC, and EC feature domains. All classifiers and feature domains showed a substantial performance reduction compared to the subject-dependent results. This highlights the challenge of generalizing emotion recognition models to unseen subjects. Median accuracies remained close to the chance level, ranging from 0.23 to 0.30. The DWT yielded the highest LOSO accuracy, with an ANN of 0.30 and SVM of 0.28. FC recorded a maximum accuracy of 0.28 using RF, while EC reached 0.28, with both RF and ANN. The widespread boxplots, reflected by the large interquartile ranges and long whiskers, indicated substantial variability across subjects. This suggests that emotion-related EEG patterns differ considerably among individuals. Consequently, models trained on generalized data struggle to maintain consistent performance when applied to unseen participants, which highlights the significant impact of intersubject variability on subject-independent emotion recognition.

### 3.4. Effect of Few-Shot Subject Adaptation

A few-shot subject adaptation strategy was employed to improve the generalizability of subject-independent emotion recognition. Pretrained models obtained from the LOSO evaluation were fine-tuned using 25%, 50%, and 75% subject-specific calibration data.

Table 7 reports the classification results at each adaptation stage for the best-performing classifier in the single feature domains (DWT, FC, and EC). In the DWT domain, ANN performed best without domain adaptation, rising from 0.28 ± 0.10 at LOSO to 0.70 ± 0.18 at 75% calibration; RF was the strongest CORAL-adapted model (0.28 ± 0.07 to 0.53 ± 0.12), while ANN again led under TCA (0.23 ± 0.08 to 0.68 ± 0.08). In the FC domain, SVM was best without adaptation (0.27 ± 0.06 to 0.54 ± 0.14), RF under CORAL (0.28 ± 0.05 to 0.62 ± 0.07), and ANN under TCA (0.24 ± 0.05 to 0.77 ± 0.06). In the EC domain, KNN was the best model both without adaptation (0.27 ± 0.04 to 0.48 ± 0.11) and under CORAL (0.26 ± 0.04 to 0.67 ± 0.15), whereas ANN led under TCA (0.25 ± 0.05 to 0.74 ± 0.06). Across these feature domains, the largest single gain consistently occurred after incorporating just 25% of subject-specific calibration data. The relative benefit of domain adaptation varied by feature domain: TCA yielded the largest improvements for FC and EC, whereas plain few-shot fine-tuning without explicit alignment remained most effective for DWT.

Table 8 summarizes the corresponding results for the multidomain feature fusion strategies. For the simple fusion method, SVM achieved the best performance without adaptation (0.24 ± 0.06 to 0.69 ± 0.10), RF performed best under CORAL (0.27 ± 0.05 to 0.65 ± 0.09), and ANN achieved the highest performance under TCA (0.25 ± 0.06 to 0.60 ± 0.11). For direct concatenation, SVM again performed best without adaptation (0.26 ± 0.07 to 0.60 ± 0.16), KNN achieved the highest performance under CORAL (0.27 ± 0.04 to 0.67 ± 0.12), and ANN performed best under TCA (0.25 ± 0.07 to 0.60 ± 0.14). Overall, both fusion strategies benefited substantially from few-shot calibration, with the greatest improvement observed after incorporating 25% of subject-specific calibration data. CORAL generally provided the strongest performance for multidomain feature fusion, whereas TCA produced more moderate improvements.

### 3.5. Comparison of EEG Classification During Emotion Induction and Auditory Stimulation

To evaluate the contribution of auditory stimulation to EEG-based emotion recognition, classification performance obtained from EEG recorded during the video-based emotion induction phase was compared with that obtained from EEG recorded during the subsequent auditory stimulation phase. Both datasets were analyzed using the same DWT feature representation (top-100 selected features) and identical machine learning classification pipeline under the subject-dependent evaluation protocol. Statistical differences in classification performance between the two conditions were assessed using a paired *t*-test.

Table 9 compares the subject-dependent classification performance obtained from EEG recorded during the video-based emotion induction phase and the subsequent auditory stimulation phase using the DWT feature representation (Top-100 selected features). Across all evaluated classifiers, EEG recorded during the auditory stimulation phase consistently achieved higher classification accuracy than EEG recorded during the video-based emotion induction phase. The paired *t*-test indicated that the improvements were statistically significant for SVM, KNN, ANN, and RF (*p* < 0.05), whereas the improvement observed for XGBoost was not statistically significant (*p* = 0.226). These findings indicate that, under the predefined emotional states established by video-based emotion induction, EEG recorded during the auditory stimulation phase achieved higher classification performance than during the emotion induction phase. This observation suggests that auditory stimulation may evoke neural responses that more clearly reflect participants’ affective states, thereby improving the discriminability of EEG features for emotion recognition. However, because the present study was conducted under controlled laboratory conditions with healthy participants, further validation is required to determine whether similar improvements can be achieved in larger and more diverse populations.

### 3.6. Computational Complexity and Real-Time Feasibility

Computational latency was assessed by measuring the total processing time for feature extraction and classification to evaluate the practical feasibility of the proposed framework for real-time BCI applications. These results confirm the need to balance predictive accuracy with computational efficiency when developing practical affective BCI systems. Because the evaluation was conducted under offline conditions, the reported processing times reflect relative computational performance and offer an initial indication of the framework’s suitability for future real-time implementation.

**Figure 5:**
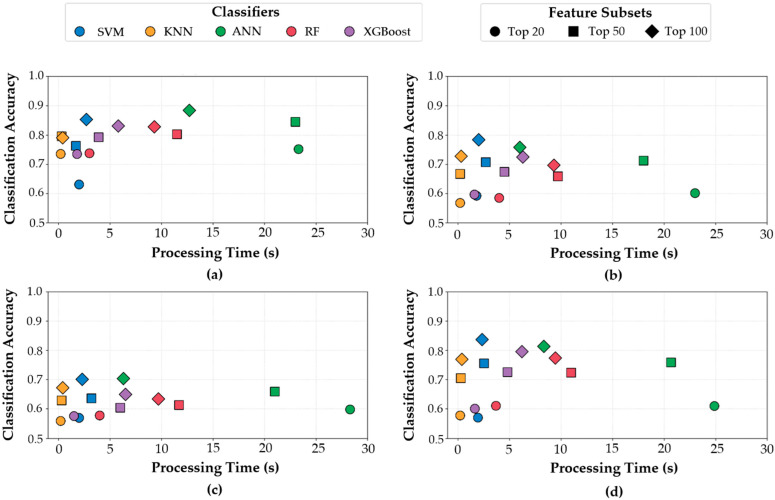
Trade-off between classification accuracy and processing time for (**a**) DWT, (**b**) functional connectivity (FC), (**c**) effective connectivity (EC), and (**d**) combined (DWT + FC + EC) feature domains. Colors indicate classifiers, whereas marker shapes denote feature subset sizes (top 20, top 50, and top 100).

Figure 5 illustrates the trade-off between classification accuracy and processing time across feature domains, classifiers, and feature subset sizes, including the combined (DWT + FC + EC) domain. The DWT features provided the highest classification accuracy, with ANN achieving 0.88 using the top-100 feature subset. The combined (DWT + FC + EC) domain achieved the second-highest accuracy (0.84 with SVM), closely approaching the DWT-only result. FC features reached a maximum accuracy of 0.78 with SVM, whereas EC features produced the lowest accuracies (up to 0.70), consistent with the subject-dependent results presented in Table 5.

Across all domains, KNN, SVM, and XGBoost remained the fastest classifiers, with processing times generally below 6 s, whereas RF required up to about 12 s. ANN was consistently the slowest and most variable classifier, with processing times ranging from about 6 s to nearly 28 s depending on the domain and feature-subset size. Overall, DWT features delivered the highest classification performance, while FC and combined features offered a favorable balance between accuracy and computational efficiency. These findings offer an initial assessment of the proposed framework’s computational characteristics and support future implementation and optimization for real-time affective BCI applications.

## 4. Discussion

EEG-based emotion recognition using auditory stimulation remains challenging because emotional responses to auditory stimuli vary widely across individuals. Inter-subject variability, together with differences in auditory perception, musical preference, and cognitive processing, may reduce the generalizability of subject-independent models. Therefore, this study systematically evaluated multiple EEG representation domains for auditory emotion recognition across subject-dependent, subject-independent, and calibration-based subject-adaptation scenarios. This study investigated whether EEG responses elicited by musical stimuli under verified emotional states provide discriminative information for auditory emotion recognition. Accordingly, the following discussion considers four aspects of the proposed framework: (1) the contribution of multidomain EEG representations, (2) the influence of inter-subject variability on subject-independent performance, (3) the effect of few-shot subject adaptation, and (4) the trade-off between classification accuracy and computational complexity.

Beyond empirical comparisons, it is important to evaluate the valence–arousal (VA) dimensional framework used in this study alongside other emotion representation methods. Previous EEG-based emotion recognition studies [17] show that emotional states can be represented either categorically, using a limited set of basic emotions such as happiness, sadness, anger, and fear, or dimensionally, by mapping emotions onto continuous axes such as valence and arousal. The categorical approach provides clear labels but may not capture blended or intermediate emotions and often assumes a fixed set of basic emotions. The dimensional VA approach, which represents emotions along two orthogonal dimensions, aligns with the four-quadrant scheme (HVHA, HVLA, LVHA, and LVLA) used in this study. However, distinguishing emotions near the center of the valence–arousal space remains challenging, and the assigned labels rely on subjective self-report rather than objective measures. In this study, predefined emotional states were induced using validated video clips before evaluating EEG responses to instrumental melodies. This design enabled a systematic investigation of auditory-evoked EEG responses under controlled affective conditions, using subject-dependent, subject-independent, and calibration-based subject-adaptation protocols.

The subject-dependent results indicate that the EEG representations evaluated captured complementary information related to emotional responses to auditory stimuli. Generally, increasing the number of selected features improved classification accuracy, implying that a subset of informative features significantly contributed to emotion recognition. Among the feature domains assessed, DWT achieved the highest accuracy, highlighting that spectral EEG data was highly informative in this study’s context. FC also performed well, suggesting that functional synchronization between brain regions provides additional valuable information. Although EC scored lower than DWT and FC, its focus on directional neural interactions may complement spectral and functional features in future research.

Table 10 compares our classification results with those reported in representative EEG-based emotion recognition studies. Under subject-dependent evaluation, DWT achieved 88.00% accuracy, while the combined feature set achieved 84.00%. These results are comparable to those reported by Wang et al. [24] (83.24–89.94%) and Cui et al. [25] (85.00%), though they are lower than the highest accuracies reported by Akhand et al. [26] (91.29–91.66%) and Zhou et al. [28] (82.37–96.47%). However, direct comparisons of classification accuracy should be interpreted with caution because the studies differ substantially in datasets, emotion induction paradigms, EEG acquisition protocols, feature representations, classification methods, and validation strategies. Most previous studies were evaluated using public benchmark datasets such as DEAP, DREAMER, and SEED, in which EEG signals were recorded during the emotion-inducing stimulus itself.

This study first established predefined emotional states using validated video clips, then examined EEG responses to instrumental melodies within these confirmed affective conditions. Unlike conventional EEG-based emotion recognition research, which typically records EEG during the primary emotion induction, this study focuses on auditory-evoked EEG responses in controlled emotional contexts. As a result, public benchmark datasets such as DEAP, DREAMER, and SEED are not directly applicable for validating this experimental paradigm. In addition, this study assessed subject-independent performance using leave-one-subject-out (LOSO) validation and calibration-based few-shot subject adaptation, in contrast to previous studies that mainly reported subject-dependent or standard cross-validation results. These evaluations offer a more comprehensive assessment of the robustness of auditory EEG representations with new subjects. Few-shot subject adaptation significantly improved cross-subject performance across all EEG representation domains. The highest classification accuracies were 0.70 for DWT without domain adaptation, 0.77 for FC using TCA, and 0.74 for EC using TCA. For multidomain feature fusion, simple fusion achieved 0.69 without domain adaptation, while direct concatenation reached 0.67 using CORAL. These results indicate that the effectiveness of subject adaptation depends on both the EEG representation domain and the feature fusion strategy.

The LOSO evaluation revealed a significant drop in accuracy across all features, nearing chance levels. This indicates that inter-subject differences greatly impacted model performance. Previous EEG emotion studies have reported similar challenges, with models trained on one group failing to generalize to new individuals, often due to physiological and cognitive differences. Our findings support this, highlighting the importance of subject adaptation for developing practical EEG-based emotion recognition systems.

The few-shot subject-adaptation experiments showed that limited subject-specific calibration substantially improved classification performance across all evaluated EEG representation domains. For DWT, FC, and EC, the largest improvement occurred after incorporating only 25% subject-specific calibration data. This indicates that a small amount of calibration data can significantly reduce inter-subject variability. As more calibration data were added, classification performance continued to improve across all feature domains.

The effectiveness of each adaptation strategy depended on the EEG representation domain. For DWT, conventional few-shot fine-tuning without explicit domain adaptation achieved the highest classification accuracy (0.70), suggesting that time–frequency representations generalize well with direct subject-specific calibration. In contrast, TCA produced the best results for FC (0.77) and EC (0.74), indicating that feature-space alignment is particularly effective for connectivity-based EEG representations. Although CORAL improved FC and EC performance, it was generally less effective than TCA for the top-performing models.

For multidomain feature fusion, both simple fusion and direct concatenation benefited from calibration-based adaptation. Simple fusion achieved the highest performance without explicit domain adaptation (0.69), while CORAL delivered the best results for direct concatenation (0.67). These results show that no single adaptation strategy is optimal for all EEG representation domains or feature fusion methods. Subject-adaptation techniques should therefore be selected based on the specific characteristics of the EEG representation.

Comparison of EEG recorded during the video-based emotion induction and auditory stimulation phases demonstrated that auditory stimulation consistently produced more discriminative EEG representations for emotion recognition across all evaluated classifiers. These findings support the hypothesis that auditory stimulation can effectively probe previously established emotional states and provide additional discriminative information for EEG-based emotion recognition.

The computational analysis identified a balance between classification performance and computational complexity. Connectivity features required more computational resources because of the intensive estimation of neural interactions, whereas the discrete wavelet transform (DWT) achieved high classification accuracy at lower computational cost. Increasing feature complexity did not consistently improve classification performance. Under the tested conditions, DWT with ANN and FC with SVM offered favorable trade-offs between accuracy and efficiency. Although this study was conducted offline with healthy participants in a controlled environment, the results demonstrate the feasibility of EEG-based emotion recognition using auditory evoked responses under verified emotional states. These findings provide practical guidance for selecting feature representations and classifiers in future research and support the advancement and clinical validation of real-time, auditory-stimulus-based affective brain–computer interface systems.

### 4.1. Limitations

Several limitations should be acknowledged:

(1) Limited participant cohort: The framework was evaluated with a small group of healthy young participants, which may restrict the generalizability of the findings to broader, more diverse populations.
(2) Emotion induction and auditory stimuli: Emotion induction used predefined affective video clips and instrumental melodies. Variability in emotional sensitivity, musical preference, and auditory perception among participants may have influenced EEG responses. Neither quantitative psychoacoustic characterization of the auditory stimuli nor formal audiometric evaluation of participants was conducted.
(3) Offline evaluation: The proposed framework was evaluated exclusively under offline conditions using pre-recorded EEG data. Consequently, its computational performance, latency, robustness, and reliability in real-time affective BCI applications remain to be established through online implementation and validation.
(4) Clinical validation: The framework has not been evaluated in clinically relevant populations, such as individuals with disorders of consciousness or communication impairments, whose neurophysiological characteristics may differ from those of healthy participants. Consequently, these findings should be regarded as evidence of feasibility under controlled laboratory conditions, serving as a basis for future validation in clinical populations and real-world settings.

### 4.2. Future Research Directions

Future research should focus on the following directions:

(1) Clinical validation: Evaluate the proposed framework in clinically relevant populations, especially patients with disorders of consciousness or communication impairments, to assess its robustness, generalizability, and clinical utility. Future studies should also better investigate the relationship between EEG-derived emotion recognition and established clinical assessment scales to understand the framework’s practical value.
(2) Larger and more diverse cohorts: Recruit larger, more heterogeneous participant populations to improve the robustness and generalizability of the framework across demographic and clinical groups.
(3) Optimization of auditory stimulation: Incorporate standardized hearing assessments and quantitative psychoacoustic characterization of auditory stimuli. Investigate additional auditory stimulation paradigms and music types to better understand their effects on emotion recognition.
(4) Adaptive and personalized learning: Develop adaptive learning approaches and subject-specific personalization strategies to reduce inter-subject variability and improve emotion recognition across subjects.
(5) Advanced deep learning models: Explore architectures that jointly learn spectral, spatial, and connectivity-based EEG representations to further improve classification accuracy and model robustness.
(6) Real-time implementation: Develop and validate an online real-time affective brain–computer interface (BCI) implementation to evaluate computational latency, classification stability, user adaptability, and practical feasibility for continuous emotion monitoring in real-world and clinical environments.
(7) Cross-dataset validation: Evaluate the proposed methodology on publicly available EEG emotion datasets where appropriate, as well as future datasets specifically designed for post-induction auditory emotion recognition, to further assess the robustness and generalizability of the framework across different experimental paradigms.
(8) Clinical applications in palliative care and disorders of consciousness: Investigate the applicability of the proposed framework for assessing emotional responses in patients receiving palliative care and in semi-conscious or minimally conscious patients who cannot communicate effectively. Future studies should explore whether EEG-derived emotional indicators can complement conventional behavioral assessments. They may assist clinicians in evaluating emotional well-being, responses to auditory stimulation, and patient-centered care.

## 5. Conclusions

This study explored EEG-based emotion recognition by analyzing auditory-evoked EEG responses under verified emotional states established using validated video clips. The DWT achieved the highest subject-dependent classification accuracy (88%), while the combined feature set (DWT + FC + EC) achieved 84%. The subject-independent (LOSO) evaluation yielded near-chance performance (approximately 30%), highlighting the influence of inter-subject variability on cross-subject emotion recognition. Few-shot subject adaptation significantly improved classification performance across all EEG representation domains. The highest accuracies were observed with DWT (70%) without domain adaptation, FC (77%) using TCA, and EC (74%) using TCA. For multidomain feature fusion, simple fusion achieved the best performance (69%) without domain adaptation, while direct concatenation reached the highest accuracy with CORAL (67%). These results indicate that the optimal adaptation strategy varies by EEG representation domain and feature fusion method. EEG recorded during auditory stimulation consistently yielded higher classification accuracy than EEG recorded during video-based emotion induction, suggesting that auditory stimulation provides more discriminative information for emotion recognition under predefined affective conditions. These findings suggest that multidomain EEG representations provide complementary information for auditory emotion recognition and that limited subject-specific calibration may improve cross-subject classification performance. The experimental paradigm demonstrates the feasibility of studying auditory-evoked EEG responses in controlled affective settings and provides a basis for developing auditory stimulus-based affective brain–computer interface systems. Future research should focus on online, real-time implementation, validation in larger and more diverse populations, advanced subject-adaptive learning strategies, and comprehensive evaluation in clinically relevant populations to establish the practical utility of the proposed framework.

## Figures and Tables

**Table 1:** Video clips selected for emotion induction based on Russell’s circumplex model of emotion.

Emotion	Symbol	Affective Quadrants	Video Code
Positive	P1	HVLA	V12
Positive	P2	HVLA	V13
Positive	P3	HVHA	V16
Positive	P4	HVHA	V18
Negative	N1	LVLA	V01
Negative	N2	LVLA	V02
Negative	N3	LVHA	V06
Negative	N4	LVHA	V09

**Table 2:** Musical stimuli categories based on Russell’s circumplex model of emotion.

No.	Stimulus Type/Name	Arousal	Valence	Symbol
M01	Instrumental melody 1	High	Positive	HVHA
M02	Instrumental melody 2	Low	Positive	HVLA
M03	Instrumental melody 3	High	Negative	LVHA
M04	Instrumental melody 4	Low	Negative	LVLA

**Table 3:** Hyperparameter configurations for machine learning models across EEG feature domains.

Model	Hyperparameters
SVM	RBF kernel, C = 10, γ = 0.01
KNN	K = 3, distance weighting
ANN	Hidden layers = (128,64), ReLU, Adam, α = 0.0001, max_iter = 500
RF	n_estimators = 200, max_depth = 20
XGBoost	n_estimators = 200, max_depth = 6, learning_rate = 0.05, subsample = 0.8, colsample_bytree = 0.8

**Table 4:** Mean subject-dependent classification accuracy obtained using RF, KNN, ANN, SVM, and XGBoost classifiers with the top 20, top 50, and top 100 selected features from the DWT, FC, EC, and combined (DWT + FC + EC) feature domains.

Feature Domain	Feature Subset	RF	KNN	ANN	SVM	XGBoost
DWT	Top 20	0.74 ± 0.06	0.74 ± 0.06	0.75 ± 0.04	0.63 ± 0.05	0.74 ± 0.05
	Top 50	0.80 ± 0.05	0.80 ± 0.06	0.85 ± 0.06	0.76 ± 0.05	0.79 ± 0.05
	Top 100	0.83 ± 0.06	0.79 ± 0.08	0.88 ± 0.05	0.85 ± 0.05	0.83 ± 0.06
FC	Top 20	0.59 ± 0.04	0.57 ± 0.03	0.60 ± 0.03	0.59 ± 0.03	0.60 ± 0.03
	Top 50	0.66 ± 0.04	0.67 ± 0.03	0.71 ± 0.03	0.71 ± 0.03	0.67 ± 0.04
	Top 100	0.70 ± 0.05	0.73 ± 0.03	0.76 ± 0.03	0.78 ± 0.03	0.73 ± 0.04
EC	Top 20	0.58 ± 0.04	0.56 ± 0.03	0.60 ± 0.03	0.57 ± 0.02	0.58 ± 0.04
	Top 50	0.61 ± 0.05	0.63 ± 0.03	0.66 ± 0.03	0.64 ± 0.03	0.60 ± 0.05
	Top 100	0.63 ± 0.05	0.67 ± 0.03	0.70 ± 0.03	0.70 ± 0.03	0.65 ± 0.05
DWT + FC + EC	Top 20	0.61 ± 0.04	0.58 ± 0.03	0.61 ± 0.05	0.57 ± 0.04	0.60 ± 0.03
	Top 50	0.72 ± 0.06	0.71 ± 0.04	0.76 ± 0.05	0.76 ± 0.04	0.73 ± 0.05
	Top 100	0.77 ± 0.06	0.77 ± 0.04	0.81 ± 0.04	0.84 ± 0.04	0.80 ± 0.05

**Table 5:** Average performance metrics (mean ± S.D.) of machine learning models across different feature domains using the top-100 feature subset for auditory stimulation.

Feature Domain	Classifier	Accuracy	Precision	Recall	F1 Score
DWT	ANN	0.88 ± 0.05	0.89 ± 0.05	0.88 ± 0.05	0.88 ± 0.05
	KNN	0.79 ± 0.08	0.79 ± 0.08	0.79 ± 0.08	0.79 ± 0.08
	RF	0.83 ± 0.06	0.83 ± 0.06	0.83 ± 0.06	0.82 ± 0.07
	SVM	0.85 ± 0.05	0.86 ± 0.05	0.85 ± 0.05	0.85 ± 0.05
	XGBoost	0.83 ± 0.06	0.83 ± 0.06	0.83 ± 0.06	0.83 ± 0.06
FC	ANN	0.76 ± 0.03	0.76 ± 0.04	0.76 ± 0.03	0.75 ± 0.04
	KNN	0.73 ± 0.03	0.73 ± 0.04	0.73 ± 0.03	0.72 ± 0.04
	RF	0.70 ± 0.05	0.70 ± 0.05	0.70 ± 0.04	0.69 ± 0.05
	SVM	0.78 ± 0.03	0.79 ± 0.04	0.78 ± 0.03	0.78 ± 0.04
	XGBoost	0.73 ± 0.04	0.73 ± 0.04	0.72 ± 0.04	0.72 ± 0.04
EC	ANN	0.70 ± 0.03	0.71 ± 0.03	0.70 ± 0.03	0.70 ± 0.03
	KNN	0.67 ± 0.03	0.67 ± 0.03	0.67 ± 0.03	0.67 ± 0.03
	RF	0.63 ± 0.05	0.64 ± 0.05	0.63 ± 0.05	0.63 ± 0.05
	SVM	0.70 ± 0.03	0.70 ± 0.03	0.70 ± 0.03	0.70 ± 0.03
	XGBoost	0.65 ± 0.05	0.65 ± 0.05	0.65 ± 0.05	0.65 ± 0.05
DWT + FC + EC	ANN	0.81 ± 0.04	0.82 ± 0.04	0.81 ± 0.04	0.81 ± 0.04
	KNN	0.77 ± 0.04	0.77 ± 0.04	0.77 ± 0.04	0.77 ± 0.04
	RF	0.77 ± 0.06	0.78 ± 0.06	0.77 ± 0.06	0.77 ± 0.06
	SVM	0.84 ± 0.04	0.84 ± 0.05	0.84 ± 0.04	0.83 ± 0.05
	XGBoost	0.80 ± 0.05	0.80 ± 0.05	0.79 ± 0.05	0.79 ± 0.05

**Table 6:** LOSO classification accuracy (mean ± SD) across feature domains and classifiers under auditory stimulation without domain adaptation.

Classifier	DWT	Functional Connectivity	Effective Connectivity	Combined (DWT + FC + EC)
RF	0.25 ± 0.05	0.28 ± 0.06	0.26 ± 0.03	0.25 ± 0.06
KNN	0.24 ± 0.05	0.25 ± 0.04	0.27 ± 0.04	0.24 ± 0.06
ANN	0.28 ± 0.10	0.27 ± 0.07	0.25 ± 0.04	0.26 ± 0.07
SVM	0.26 ± 0.06	0.27 ± 0.06	0.27 ± 0.03	0.26 ± 0.07
XGBoost	0.26 ± 0.05	0.29 ± 0.06	0.26 ± 0.04	0.26 ± 0.06

**Table 7:** Classification accuracy obtained under LOSO and few-shot adaptation settings for the best-performing classifier in each feature domain.

Feature Domain	Best Classifier	Adaptation	Zero-Shot (LOSO)	Few-Shot (25%)	Few-Shot (50%)	Few-Shot (75%)
DWT	ANN	No Adaptation	0.28 ± 0.10	0.40 ± 0.08	0.53 ± 0.16	0.70 ± 0.18
	RF	CORAL	0.28 ± 0.07	0.42 ± 0.08	0.49 ± 0.09	0.53 ± 0.12
	ANN	TCA	0.23 ± 0.08	0.51 ± 0.08	0.60 ± 0.08	0.68 ± 0.08
FC	SVM	No Adaptation	0.27 ± 0.06	0.39 ± 0.08	0.48 ± 0.11	0.54 ± 0.14
	RF	CORAL	0.28 ± 0.05	0.45 ± 0.04	0.53 ± 0.05	0.62 ± 0.07
	ANN	TCA	0.24 ± 0.05	0.62 ± 0.07	0.70 ± 0.07	0.77 ± 0.06
EC	KNN	No Adaptation	0.27 ± 0.04	0.36 ± 0.06	0.40 ± 0.10	0.48 ± 0.11
	KNN	CORAL	0.26 ± 0.04	0.44 ± 0.06	0.56 ± 0.11	0.67 ± 0.15
	ANN	TCA	0.25 ± 0.05	0.58 ± 0.06	0.68 ± 0.07	0.74 ± 0.06

**Table 8:** Classification accuracy obtained under LOSO and few-shot adaptation settings for the best-performing classifier in multidomain feature.

Fusion Method	Best Classifier	Adaptation	Zero-Shot (LOSO)	Few-Shot (25%)	Few-Shot (50%)	Few-Shot (75%)
Simple	SVM	No Adaptation	0.24 ± 0.06	0.41 ± 0.08	0.51 ± 0.09	0.69 ± 0.10
	RF	CORAL	0.27 ± 0.05	0.43 ± 0.08	0.54 ± 0.07	0.65 ± 0.09
	ANN	TCA	0.25 ± 0.06	0.41 ± 0.09	0.52 ± 0.08	0.60 ± 0.11
Direct Concatenation	SVM	No Adaptation	0.26 ± 0.07	0.41 ± 0.08	0.51 ± 0.10	0.60 ± 0.16
	KNN	CORAL	0.27 ± 0.04	0.43 ± 0.03	0.55 ± 0.08	0.67 ± 0.12
	ANN	TCA	0.25 ± 0.07	0.46 ± 0.11	0.55 ± 0.09	0.60 ± 0.14

**Table 9:** Subject-dependent classification accuracy (mean ± SD) comparing EEG recorded during the video-based emotion induction phase and the auditory stimulation phase using the DWT feature representation (top-100 selected features).

Classifier	Video-Based Emotion Induction	Auditory Stimulation	*p*-Value
SVM	0.79 ± 0.10	0.88 ± 0.05	0.037
KNN	0.65 ± 0.10	0.79 ± 0.08	0.011
ANN	0.71 ± 0.07	0.83 ± 0.06	0.009
RF	0.73 ± 0.10	0.85 ± 0.05	0.012
XGBoost	0.79 ± 0.09	0.83 ± 0.06	0.226

**Table 10:** Comparison of classification accuracy between this study and representative previous EEG-based emotion recognition studies.

Study	Stimuli	Datasets	Emotion	Features	Methods	Validation	Accuracy (%)
Li et al. [23]	Affective videos	Self-collected HI	Positive–negative	DE maps + facial images	CBAM-ResNet34	Subject-dependent split	78.32
Wang et al. [24]	Music and videos	DEAP, DREAME, SEED	Valence–arousal	PLV networks + PH features	SVM	Repeated 80/20 hold-out	DEAP: 89.94; DREAMER: 87.61; SEED: 83.24
Cui et al. [25]	Music videos	DEAP	Valence–arousal	PLV adjacency matrices	CNN	Individual/group comparison	85.00
Akhand et al. [26]	Music videos	DEAP	Valence–arousal	PCC, PLV, MI, TE maps	CNN	5-fold CV	Valence: 91.29; Arousal: 91.66
Wei et al. [27]	Music	Self-collected MDD/HC	Clinical classification	Delta-band DFC	Ensemble classifier	LOSO-CV	86.10
Zhou et al. [28]	Music videos and audio	DEAP, DREAMER, and music	Valence–arousal	PSD + MIR features	EER/MEC + LIBSVM	10-fold CV	DEAP: 96.47; DREAMER: 82.37; Music: 94.00%
Bo et al. [29]	Music clips	Self-collected EEG	Valence–arousal	Temporal–spectral BPC	SVM	Binary and three-class evaluation	Valence: 66.80; Arousal: 59.50
This study	Affective videos + instrumental melodies	Self-collected EEG	Valence–arousal	DWT, FC, EC	ANN, KNN, RF, SVM, XGBoost	Subject-dependent (10-fold), LOSO, and few-shot adaptation	Subject-dependent (DWT): 88.00; LOSO: ~30.00; Few-shot (FC, 75% calibration): 77.00

Table Note: HI = hearing impaired; DE = differential entropy; PLV = phase-locking value; PH = persistent homology; PCC = Pearson correlation coefficient; MI = mutual information; TE = transfer entropy; DFC = directed functional connectivity; PSD = power spectral density; MIR = mutual information rate; DWT = discrete wavelet transform; FC = functional connectivity; EC = effective connectivity; CNN = convolutional neural network; ANN = artificial neural network; RF = random forest; KNN = k-nearest neighbors; SVM = support vector machine; LIBSVM = Library for Support Vector Machines; LOSO-CV = leave-one-subject-out cross-validation; MDD = major depressive disorder; HC = healthy control.

## Data Availability

The data presented in this study are available upon request from the corresponding author.

## Abbreviations

The following abbreviations are used in this manuscript:

ANN	Artificial neural network
ANOVA	One-way analysis of variance
BCI	Brain–computer interface
CNN	Convolutional neural network
DE	Differential entropy
DWT	Discrete wavelet transform
EC	Effective connectivity
EEG	Electroencephalography
EER	Enhanced entity-relationship
FC	Functional connectivity
FDR	False discovery rate
HC	Healthy control
HI	Hearing impaired
HVHA	High valence–high arousal
HVLA	High valence–low arousal
KNN	k-nearest neighbors
LOSO	Leave-one-subject-out
LVHA	Low valence–high arousal
LVLA	Low valence–low arousal
MDD	Major depressive disorder
MFCC	Mel-frequency cepstral coefficient
MI	Mutual information
MIR	Music information retrieval
PCC	Pearson correlation coefficient
PLV	Phase-locking value
PSD	Power spectral density
RBF	Radial basis function
RF	Random forest
SVM	Support vector machine
TE	Transfer entropy
XGBoost	Extreme gradient boosting
TCA	Transfer component analysis
CORAL	Correlation alignment
